# Medico-Legal Implications and Risk Management Strategies in Orthodontic Practice: An Analytical Literature Review

**DOI:** 10.3390/healthcare13233054

**Published:** 2025-11-25

**Authors:** Flavius Costanza-Gugiu, Ana Cernega, Silviu-Mirel Pițuru

**Affiliations:** Department of Organization, Professional Legislation and Management of the Dental Office, Faculty of Dental Medicine, “Carol Davila” University of Medicine and Pharmacy, 17-23 Plevnei Street, 020021 Bucharest, Romania; flavius.costanza-gugiu@drd.umfcd.ro (F.C.-G.); silviu.pituru@umfcd.ro (S.-M.P.)

**Keywords:** orthodontics, malpractice, risk management, liability, medico-legal, law-suit, vuca, doctor–patient relationship

## Abstract

**Introduction:** Orthodontic practice, though often considered low-risk compared with other dental specialties, is increasingly exposed to medico-legal challenges shaped by the volatile, uncertain, complex, and ambiguous (VUCA) environment of modern healthcare. The aim of this review was to identify the underlying causes and typologies of malpractice claims in orthodontics. **Methods:** This narrative review synthesizes evidence from 2010–2024 on malpractice allegations and risk management strategies in orthodontics, with attention to the clinical, ethical, and legal dimensions. **Results:** The analysis of the included articles identified the most common types of malpractice allegations and the share of malpractice allegations of orthodontists in relation to other specialties; the main risks of orthodontic practice were identified, as well as risk management strategies in orthodontics. The findings highlight that most malpractice allegations are not related to medical/clinical errors, but to ethical–legislative, behavioral/relational, and perceptual/aesthetic vulnerabilities. **Conclusions:** This review highlights the need for physicians to focus on the acquisition of transversal skills, to have the ability to understand, adapt and respond to the rational and emotional needs of patients. This is essential in an ever-changing world, supporting healthcare professionals in maintaining professional safety. This review opens a relevant research direction on the role and impact of digital technology in the orthodontist–patient relationship, having the ability to influence the dynamics of this therapeutic relationship and the perception of the treatment outcome.

## 1. Introduction

Orthodontics and Dento-Facial Orthopedics is the specialty of dentistry that deals with the supervision, guidance and correction of the dento-facial structures of the body, whether growing or mature [1]. The interest in this specialty is growing, both due to the fact that patients have become aware that oral health is closely related to systemic health, and due to the aesthetic rigors of the society in which we live [1]. Currently, Orthodontics and Dento-Facial Orthopedics is in a continuous process of evolution and transformation, whether we are talking about the increasingly varied therapeutic arsenal, the more precise investigation methods, the digitization of certain aspects of practice or the use of artificial intelligence (AI) as an adjunct to orthodontic treatments.

This specialty has several traits that provide a specific perspective of it. As for orthodontic treatments, they usually extend over a long period of time, which considerably amplifies the volatile size of the associated costs. This financial component borne by the patient contributes to increasing expectations regarding the results of orthodontic treatment. Moreover, the complexity of the doctor–patient relationship is induced by the existing informational imbalance: the low level of medical knowledge guides the patient to request orthodontic treatments more for aesthetic than functional reasons, making it more difficult for the doctor to explain that the function of the dento-maxillary apparatus post-treatment is the primary role of this specialty. In this context, the ambiguity derives from the disproportionate assessment of what the success of orthodontic treatment means, an aspect that may represent the basis of litigious actions. These features specific to this specialty prove the existence and influence of the VUCA context (volatility, uncertainty, complexity and ambiguity), which generates instability and unpredictability within the professional medical activity [2]. VUCA influences the healthcare field, including orthodontic practice, by amplifying the reputational and legal risks of orthodontists.

Thus, despite the marked evolution of this specialty in recent decades, just like any other medical specialty, Orthodontics and Dentofacial Orthopedics is no exception to the risks and vulnerabilities associated with the practice, whether we are talking about the doctor or the patient. We are talking about both the doctor and the patient as both represent the main actors of the medical system, and the doctor–patient relationship represents the foundation of the health system [2].

The importance of this relationship lies in the respect of fundamental principles of modern society, namely, respect for the right to life and health, which are an integral part of the Declaration of Human Rights [3]. Moreover, this relationship represents the origin of all legal interactions established in the context of healthcare services delivery (doctor–patient, doctor–doctor, doctor–healthcare institution, patient–healthcare institution) [2]. Thus, to protect these relationships, numerous legal frameworks and normative acts have been established that clearly outline the rights of patients and the corresponding obligations of medical professionals. Non-compliance with these legally defined provisions undermines the integrity of the doctor–patient relationship and often prompts patients to seek legal recourse and financial compensation.

The fact is that we live in a society where the number of disputes is constantly increasing, a society in which autonomous patients have access to more information, increases their expectations regarding orthodontic treatments and decreases their tolerance for professional errors [4]. Although orthodontics is generally associated with lower litigation rates (5.1%) compared to other dental specialties, there remains a notable gap in systematic analyses addressing medico-legal risks specific to orthodontic practice [5]. To address this gap, we conducted a literature review to answer the following research question: *What are the most frequently reported types of clinical errors, procedural issues, or professional vulnerabilities associated with malpractice claims in orthodontic practice, according to the scientific and medico-legal literature?”*.

## 2. Materials and Methods

This narrative review was conducted to synthesize the medico-legal vulnerabilities and risk management strategies reported in orthodontic practice. A literature search was carried out in PubMed, Web of Science, ScienceDirect, and Google Scholar, covering articles published in English between 2010 and 2024. The last 15 years were chosen as relevant due to changes in legislation and technology. Both original research and expert commentaries were included to capture clinical, ethical, and legal perspectives relevant to orthodontics.

The literature was thematically analyzed and organized into categories reflecting the most common types of malpractice allegations; the share of malpractice allegations of orthodontists in relation to other specialties; identification of the main risks of orthodontic practice; and the risk management strategies in orthodontics.

Studies were included in this review based on the following criteria:

- *Language and publication date:* Only articles published in English between 2010 and 2024 were considered. This time interval was selected to ensure relevance to current orthodontic practices and legal frameworks, considering the increasing complexity of medico-legal environments and the evolution of digital technologies in orthodontics.
- *Topic relevance:* Eligible studies explicitly addressed medico-legal implications, liability, malpractice, or risk management strategies in orthodontic practice. This includes studies that
  ◦ Analyze civil lawsuits or malpractice claims related to orthodontic procedures;
  ◦ Discuss clinical or administrative risk factors leading to litigation;
  ◦ Explore communication, ethical, or consent-related challenges with legal consequences;
  ◦ Propose strategies or tools to reduce medico-legal vulnerability in orthodontics.
- *Types of studies:* The following types of publications were eligible:
  ◦ Original research articles (qualitative, quantitative, or mixed-methods studies);
  ◦ Retrospective analyses of legal or insurance databases;
  ◦ Systematic reviews or narrative reviews with legal relevance;
  ◦ Case reports, case series, or expert commentaries that provide insight into litigation or medico-legal disputes in orthodontics;
- *Population:* Studies had to focus specifically on orthodontic practice, including pediatric or adult orthodontics. Articles centered on general dentistry or other dental specialties (e.g., prosthodontics, periodontics, endodontics) were excluded unless they provided comparative data that included orthodontics.

Articles were excluded from the review if they met any of the following conditions:

- Non-English publications, or abstracts without full-text availability;
- Published before 2010, unless they were landmark legal studies still referenced widely in recent literature (none were retained);
- Studies that addressed general dentistry or other specialties (e.g., prosthodontics, oral surgery) without specific reference to orthodontics;
- Articles focusing exclusively on clinical techniques or biomechanical outcomes, without any reference to risk management, legal, ethical, or liability issues.

## 3. Results

Table 1 presents in summarized form information regarding the articles analyzed from the point of view of the authors, the year of publication, the country, and the type of subject analyzed, this being our starting point in terms of the analysis we have made.

### 3.1. Types of Malpractice Allegations in Orthodontics

An article published in 2012 by Franco et al., which analyzed 108 civil lawsuits in Brazil between 2003 and 2009, shows that more than half of the malpractice allegations were represented by unsatisfactory treatment results (32.41%), as well as by the intervention of another orthodontist (25%) following the request for a second opinion by the patient [8]. Other reasons justifying the accusations of malpractice were represented by oral lesions (20.37%) and too long treatment time (12.96%) [8].

Another study published in 2020 by Picoli et al. analyzed all civil court lawsuits in Brazil from 1998 to 2017, which were related to orthodontics [16]. Of the 395 civil lawsuits analyzed, 337 (85.3%) were judged between 2011 and 2017, highlighting a sharp increase in orthodontic litigation during this period [16]. The main cause of lawsuits filed against orthodontists was patients’ dissatisfaction with the results of orthodontic treatment, reported in 143 cases (36.2%), followed by periodontal disease in 45 cases (11.4%) and the need for tooth extractions during treatment in 43 cases (10.9%). Regarding the duration of orthodontic treatment, only 52.9% of the analyzed disputes provided such information, with an average duration of 3.9 years, with extremes ranging from 0.3 years to 14 years [16].

In a 2014 study supported by the Korean Association of Orthodontics, Kim et al. analyzed medical disputes from both patient and clinician perspectives [16]. The primary causes of litigation were dissatisfaction with the results of the treatment (18.6%), followed by root resorptions (9.7%), pulp necrosis (8.3%) and temporomandibular dysfunctions (7.6%) [9]. The study also explored perceived triggers of conflict: nearly half of the patients (48.4%) cited clinician negligence, followed by inadequate explanations (32%), cost-related disagreements (7.3%), and dissatisfaction with final results (5.3%) [9]. In contrast, orthodontists identified patient misunderstanding (39.7%) and psychological issues (29.8%) as the main contributors to conflict [9]. The study also examined the influence of clinician experience and patient age on the likelihood of conflict [9]. A significant association was observed between the duration of orthodontic practice and the incidence of medical accidents: 63.6% of litigation cases involved orthodontists with over 10 years of experience, followed by those with 5–10 years (23.2%) and less than 3 years (6.1%) [9]. Regarding patient demographics, the majority of incidents involved adults—47.3% of affected patients were over 25 years old, with decreasing frequencies among younger age groups: 21.8% (19–24 years), 20.2% (13–18 years), and 10.6% (under 12 years). These findings suggest that medico-legal disputes are more prevalent among experienced practitioners and adult patients [9].

A study conducted in Israel by Laviv et al. analyzed all orthodontic-related complaints filed between 2005 and 2018, specifically including cases involving periodontal disease [15]. Of over 1500 complaints related to periodontal pathology during this period, only 35 cases (2.3%) were associated with orthodontic treatment [15]. Patients who pursued legal action most commonly reported worsening of periodontal disease (97.1%), dental injuries or tooth loss (62.9%), compromised esthetics (57.1%), and pain or discomfort (42.9%) [15]. The main allegations in litigation included delayed initiation of orthodontic treatment (97.1%), treatment undertaken in the presence of active periodontal disease (91.4%), and failure to diagnose existing periodontal conditions (54.3%) [15]. The average age of affected patients was 23.4 years (range: 12–51 years), and the mean treatment duration was 3.2 years [15]. In a study endorsed by the American Association of Orthodontists (AAO), Pour et al. distributed an electronic questionnaire to a random sample of 2241 active AAO members to investigate the most common causes of malpractice complaints in orthodontics [18]. Among the 77 orthodontists who responded, (9.1%) reported having been the subject of a malpractice claim [18]. The leading cause was periodontal complications (3 cases), followed by isolated cases involving root resorption, a failed attempt to extrude an impacted and ankylosed tooth, ethical conflicts between colleagues, and tooth loss due to combined periodontal and endodontic pathology [18].

When asked to rank contributing factors to malpractice claims, respondents identified the breakdown of the doctor–patient relationship as the most significant factor (58.4%), followed by lack of informed consent (30.3%) and periodontal issues (24.7%) [18].

All reported causes of malpractice allegations identified across the included studies are summarized in Table 2, providing an overview of the most frequently cited clinical, communicational, and ethical factors.

### 3.2. The Share of Malpractice Accusations of Orthodontists in Relation to Other Specialties

Studies conducted in South Africa and Brazil highlighted a low share of complaints for malpractice in orthodontics, reporting values of 4.5% and 8.8%, respectively, of the total cases analyzed [14,20]. In contrast, research conducted in Saudi Arabia indicated a much higher frequency, with 20.2% of malpractice cases reported in orthodontics, placing this specialty in second place, after dental prosthetics [17]. These findings are summarized in Table 3.

Despite the higher incidence of malpractice claims in Saudi Arabia, the comparative analysis across countries and dental specialties offers a nuanced perspective on the medico-legal risk associated with orthodontic practice. These variations suggest that the orthodontic doctor–patient relationship may exhibit distinct characteristics, influenced by specific patient expectations, extended treatment durations, and unique perceptions of treatment outcomes, as previously discussed in this review.

### 3.3. The Main Risks of Orthodontic Practice

In the context of the data presented above, risks and complications in orthodontic practice refer to treatment-specific adverse outcomes that may negatively impact the patient. Once such complications are perceived or identified, they may prompt the patient to pursue legal action, generating legislative consequences that can affect the responsible clinician both professionally and reputationally.

To systematize the main complications and medical risks associated with orthodontic treatments, we will use the following classification [12]:

(a) *Dental complications:* demineralizations, caries, dental wear, enamel cracks or fractures, and dyschromia. At the root level, resorption, premature closure of the apex or ankylosis may occur, and at the level of the dental pulp, ischemia, pulpitis or even necrosis.
(b) *Periodontal complications:* gingivitis, periodontitis, gingival hypertrophy, gingival retractions, bone resorption, dehiscences, fenestrations or black triangles.
(c) *Soft tissue changes*, such as damage to the gingival or oral mucosa, chemical or thermal burns. Stomatitis, gingival hyperplasia around the brackets or ulcerations may also occur.
(d) *Temporomandibular joint dysfunctions*.
(e) *Unsatisfactory treatment outcomes* both functional and aesthetic, or relapse [12].

Other risks not included in this classification, though relatively infrequent, warrant consideration due to their potential severity. These include accidental ingestion or aspiration of small orthodontic components and allergic reactions to materials used during treatment [7,10].

At the same time, there are a number of risky actions that can make the orthodontist vulnerable in the event of a dispute, such as: ***inadequate clinical assessment***, which can lead to misdiagnosis and/or improper treatment planning, ***failure to recognize pre-existing conditions*** and ***the lack of informed consent* [10]**.

### 3.4. Risk Management Strategies

The data presented in the previous sections confirm that the risk of error in orthodontic treatment is real and shaped by the specific characteristics of the specialty. Such risks can lead to litigation, directly affecting the clinician’s professional security. In this context, understanding risk management mechanisms and developing effective strategies for the orthodontist–patient relationship becomes essential. This is particularly important given that healthcare is a domain characterized by zero tolerance for error—an expectation justified by the inherent fragility of human life and well-being. To better systematize the risk management strategies identified in the literature, we propose analyzing the doctor–patient relationship across the three essential phases of orthodontic care: pre-treatment, treatment, and post-treatment [10]. By identifying and understanding the specific vulnerabilities associated with each phase, targeted interventions can be implemented to reduce conflict and strengthen the therapeutic alliance.

#### 3.4.1. Pre-Treatment Phase

The pre-treatment phase is the moment of the doctor’s first contact with the patient. We will mainly address the medico-legislative aspects of the practice, we will emphasize the importance of building a strong doctor–patient relationship, based on trust, but also the need to perform a comprehensive clinical examination and all necessary investigations.

At the first consultation, the patient must be ***informed*** on the diagnosis, mentioning the possible procedures that will be involved, explaining the importance of performing quality investigations, as well as estimating the costs and presenting the different orthodontic appliance systems [6]. It is also important to identify any pre-existing pathologies, to properly document them, but also to request clearance from the general dentist, pedodontist or periodontist for orthodontic treatment, forming an ***interdisciplinary team*** to minimize the potential risks [4,6].

Another key aspect that must be emphasized during the first consultation is ***communication***. Clinicians should use language that is age-appropriate and tailored to the patient’s level of understanding, ensuring that all relevant information is conveyed in an accessible and comprehensible manner [6,10]. It is also important to understand the patient’s motivation, to try a realistic scaling of his expectations from the beginning, emphasizing the main purpose and limitations of orthodontic treatment, without making promises or offering guarantees [4,6].

Discussing the case and presenting the treatment plan is arguably the most critical stage of the entire doctor–patient interaction [6]. This is the moment when **informed consent** is obtained, marking the legal and ethical foundation of the therapeutic relationship [6]. Patients should be presented with all viable treatment options—including the option of no treatment—along with the associated risks and benefits of each approach, to facilitate an autonomous, well-informed decision [4,10]. Additionally, it is essential to explain the concept of retention and provide transparent information regarding the costs associated with each phase of treatment [6].

In contemporary orthodontics, the growing demand for treatment among adult patients—who often present with more complex cases and higher aesthetic expectations than adolescents—places increased emphasis on effective, personalized risk communication [19]. Tailoring discussions to the psychological profile of the adult patient is a central component of risk management [19]. Integrating psychosocial factors such as anxiety, depression, or perfectionism into the therapeutic dialogue enhances both treatment adherence and the establishment of a doctor–patient relationship rooted in trust and collaboration [19].

According to Zhao et al., adult patients perceive ***negative changes in facial appearance*** as the most significant treatment-related risk [19]. It is therefore essential to explain that certain age-related aesthetic changes—such as mild profile flattening—are natural outcomes of the continuous mandibular growth into adulthood, independent of whether extractions are performed [10,19].

#### 3.4.2. The Active Treatment Phase

In the active phase of the orthodontic treatment, it is important to ***monitor progress***, always keeping in mind the end goals and control of the duration of treatment [6]. Whether or not complications arise during treatment, it is essential for the clinician to keep clear medical records, in analogue or digital format, both for the quality of the medical act and for its protection in the event of a dispute [6]. At each appointment, records should be made of all medical procedures performed, but also of unwanted side effects (pain, tooth mobility, gingival inflammation) or those caused by the patient’s lack of cooperation and poor oral hygiene (demineralization lesions, detachment of certain elements of the appliance, failure to attend appointments or non-compliance with the doctor’s instructions) [6].

During the active phase of orthodontic treatment, it is essential to monitor progress consistently, maintaining focus on both the treatment objectives and the overall duration of therapy [6]. Regardless of whether complications arise, the clinician must maintain detailed medical records, not only to ensure the quality of care, but also to provide legal protection in the event of a dispute [6]. At each appointment, records should include all procedures performed, as well as any adverse events such as pain, increased tooth mobility, or gingival inflammation [6]. Patient-related issues, including poor oral hygiene, missed appointments, or failure to comply with instructions must also be thoroughly recorded [6].

Given the high prevalence of white spot lesions (up to 70%), carious lesions (approximately 5%), and demineralization of the maxillary incisors (over 30%) during orthodontic treatment, the implementation of effective prevention strategies is essential [12]. Primary prevention measures, including patient education on oral hygiene and dietary habits, should be supported by the use of fluoride-releasing bonding materials and professional topical fluoridation [4,12]. Concurrently, secondary prevention through the daily use of fluoridated products, such as toothpaste and mouth rinses, plays a critical role in reducing the risk of caries throughout orthodontic therapy [10,12].

Oral hygiene is essential in minimizing gingival hyperplasia, a common complication of orthodontic treatment with an estimated prevalence of 10% [13]. Brackets and elastics contribute to bacterial plaque accumulation, often leading to gingival inflammation in patients with inadequate oral hygiene [10,13]. Gingival inflammation around orthodontic bands can result in the formation of pseudo-periodontal pockets and loss of alveolar attachment, particularly in the distal segments of the arches [13].

Given that orthodontic bands increase plaque retention, hinder proper hygiene (e.g., flossing), and carry risks such as soft tissue injury and discomfort during placement, the use of tubes is recommended instead [4,13]. Throughout treatment, it is essential to monitor patients’ oral hygiene and reinforce motivation [4,13]. In cases of persistent non-compliance, it may be clinically appropriate to reassess the treatment plan and consider early termination to prevent irreversible damage that could outweigh the benefits [4,6].

Root resorption is an inevitable consequence of orthodontic treatment, with a prevalence of 100% in studies performed under the microscope and can occur in varying degrees [7,17]. Although root resorptions larger than 4 mm occur with a rather low frequency (1–6.6%), it is very important to perform ***panoramic X-rays*** 9 months after treatment initiation [4,10,12].

Another important consideration during treatment is the potential detachment and loss of orthodontic appliance components [6]. These events must be documented and carefully monitored, with attention to whether the dislodged elements have been expelled, ingested, or aspirated [6]. Ingestion of dental foreign bodies has an incidence ranging from 3.6% to 27.7%, therefore both general and specific management strategies are needed for such situations [6].

As for the general management strategies for such situations, the patient will be positioned vertically, rather than horizontally, when risky maneuvers are performed [7]. At each visit, clinicians must check the integrity of all appliances and advise patients not to attempt self-repair, but to contact the orthodontist immediately in case of detachment [12]. Equally important is that both orthodontists and clinic staff should be trained in first aid, with knowledge refreshed at least every two years [7].

There are a number of specific recommendations for the prevention of these cases when fixed orthodontic appliances are used: securing bands, quadhelixes and transpalatal arches with dental floss, additional reinforcement of the place where they are attached with metal ligatures and periodic checking of the cutting instruments [7]. High-quality materials and instruments should be used consistently, and replaced at any signs of wear or fatigue [7,11].

As part of orthodontic risk assessment, the potential for allergic reactions to materials must be considered. While intraoral nickel allergies are rare, extraoral manifestations are more frequent, particularly with the use of extraoral appliances containing metal components [10]. Identifying any allergic sensitivities during the initial consultation is essential to prevent complications throughout long-term orthodontic treatment [10].

Before appliance removal and transitioning to the post-treatment phase, patients should be given the opportunity to evaluate the final tooth position [6]. This step allows the orthodontist to confirm patient satisfaction and reinforces a positive conclusion to the therapeutic relationship [6]. If the treatment outcome differs from the initial plan, it is crucial to communicate and explain this directly to the patient, thereby minimizing the risk of dissatisfaction or future conflict, especially if the issue is later highlighted by another practitioner [6].

During debonding, brackets should remain attached to the archwire to minimize the risk of ingestion or aspiration [7]. Extra caution is required when removing ceramic brackets, as they are prone to fracture and may cause enamel damage [6,10]. Although enamel loss ranging from 0 to 30 microns is considered inevitable following orthodontic treatment, patients should be informed in advance about these potential risks of esthetic brackets [10].

#### 3.4.3. Post-Treatment Phase

In the post-treatment phase, it is essential to revisit the concept of retention, including its limitations and the factors influencing long-term stability (unerupted third molars or mandibular growth [6]. Patients should understand that alveolar bone remodeling continues throughout life, and minor post-treatment tooth movements do not necessarily indicate relapse [4]. Given the varying relapse potential of different malocclusions, the orthodontist must select an appropriate retention type and duration, while also explaining the associated oral hygiene requirements [4,6].

## 4. Discussion

### 4.1. Typology of Conflicts in Orthodontics

Despite the limited number of studies, clinical risks in orthodontic practice are well documented, as are the strategies for their management. However, a comparative analysis reveals a significant discrepancy: most malpractice allegations are not directly linked to clinical errors, but to adjacent issues. These can be categorized into three main vulnerability areas:

1. ***ethical—legislative vulnerabilities***—such as lack of clear informed consent, poor documentation of risk factors, and failure to inform patients of potential complications.
2. ***behavioral—relational vulnerabilities***—including poor communication, unmet expectations, and a lack of doctor–patient alignment.
3. ***perceptual—aesthetic vulnerabilities***—often stemming from misaligned expectations regarding treatment duration, goals, and outcomes.

These vulnerabilities often relate more to emotional and social dynamics than to strictly medical failures. Risk management, therefore, must include strategies that go beyond clinical precision—specifically, the development of emotional intelligence and effective communication. These skills help bridge the gap between functional treatment outcomes and patient expectations, enhance trust, and reduce litigation risks. While their presence often goes unnoticed, their absence is a major trigger for conflict in orthodontic care.

From a risk management perspective, certain tools lie within the orthodontist’s direct control—most notably, the development of emotional intelligence and effective communication skills. These transversal competencies are essential not only for preventing automatism in clinical behavior, but also for fostering trust-based, empathetic doctor–patient relationships. Emotional intelligence, understood as the capacity to recognize and regulate one’s own emotions while understanding those of others, supports relational balance. Communication complements this by bridging the gap between the patient’s expectations and the functional realities of treatment. Effective communication offers distinct benefits:

- *From the **patient**’s point of view*—it clarifies medical reasoning, sets realistic expectations, supports autonomy, and enhances adherence.
- *From the **clinician**’s point of view*—it builds trust, reduces misunderstandings, improves efficiency, and lowers reputational and legal risk.

These emotional elements are often invisible when functioning well, yet their absence becomes sharply apparent—sometimes severe enough to lead to litigation. In this sense, communication acts as a psychological safeguard within the orthodontic relationship, mitigating conflict before it arises.

### 4.2. Effective Risk Management Strategies in Contemporary Orthodontics

Risk management in orthodontic practice is also influenced by the organizational structure of the clinical setting. Larger practices tend to maintain stricter adherence to standardized protocols, supported by internal audits and hierarchical communication systems that reduce ambiguity in decision-making. Conversely, in smaller or individual practices, the closer and more informal relationship between practitioner and patient can blur professional boundaries, potentially allowing patient involvement in clinical decisions and leading to dissatisfaction or conflict when such involvement is restricted.

Despite significant technological advancements in orthodontics, none of the reviewed articles on risk management strategies addressed the integration of digital tools into clinical practice. This omission is notable given the increasing relevance of digitalization in today’s VUCA world. The incorporation of digital technologies not only enhances the predictability and precision of treatment but also serves as a valuable tool for improving doctor–patient communication—thereby potentially reducing the risk of conflict and increasing the overall quality of care [21].

A recent systematic review by Alam et al. (2023) underscores the rapid digital transformation of orthodontics, driven by emerging technologies such as artificial intelligence, 3D printing, aligners, and teleorthodontics [22]. These innovations enhance diagnostic precision and therapeutic efficiency, while also improving personalization, accessibility, and care continuity, particularly in restrictive contexts like the COVID-19 pandemic [22]. However, the authors also call for caution, highlighting the importance of further research to validate clinical efficacy and to address potential ethical and medico-legal risks associated with the adoption of these technologies [22].

Despite requiring substantial investment in equipment and staff training, the integration of digital technologies in orthodontics enhances clinical efficiency, shortens treatment duration, and improves doctor–patient communication, particularly in complex interdisciplinary cases, increasing both treatment acceptance and patient compliance [23,24].

Interestingly, while aesthetic alternatives such as ceramic or lingual brackets have seen limited success, the last decade has witnessed an exponential rise in aligner treatments, with the global market valued at USD 3.1 billion in 2021 and projected to reach USD 11.6 billion by 2027, reflecting a compound annual growth rate (CAGR) of 13% [25].

The clear aligner market is expected to continue its rapid expansion in the coming years, accompanied by a growing number of general dentists providing orthodontic treatments with aligners. Recent studies confirm this trend, reporting that 65% of general dentists in Australia already offer clear aligner therapy, with most others intending to adopt it in the near future [26]. Similarly, a European survey found that 79% of practitioners—83% of orthodontists and 65% of general dentists—use clear aligners in their practice, and 69% of non-users plan to implement them [27]. Notably, 45% of orthodontists acknowledge that clear aligner treatments may limit therapeutic outcomes, compared to only 5% of general dentists, even though 40% of the general dentists reported not having experience with this type of appliance [27].

While this widespread adoption reflects the increasing popularity and accessibility of aligner therapy, it also raises concerns about potential clinical errors and patient dissatisfaction. The question remains whether general dentists can adequately address the functional aspects of orthodontic treatment, or merely the aesthetic ones, as they may lack the biomechanical understanding of the orthodontic treatments, essential for successful outcomes [28].

Aligner treatments represent a unique therapeutic option, distinguished by significantly higher patient acceptance compared to fixed appliances. This can be largely attributed to the emotional needs of the patient, which aligners address effectively. They support the ***need for affiliation***, by allowing discreet integration into social contexts without aesthetic compromise; the ***need for recognition***, through their association with cutting-edge technology; the ***need for control***, by enabling patients to actively manage their treatment; and the ***need for safety***, through transparent and predictable treatment planning.

Yet, a critical question remains: Can aligner treatments, with current materials and biomechanics, deliver functional outcomes across all malocclusion complexities—or are they primarily a response to aesthetic desires? This question may serve as a valuable direction for future research, potentially reframing the way orthodontists approach therapeutic planning and doctor–patient communication.

Furthermore, as discussed earlier, patients’ expectations are increasingly aesthetic in nature, which often clashes with the orthodontist’s focus on functional correction. This disconnect is a frequent source of conflict in clinical practice. In this context, the integration of ***AI and digital tools***—especially as instruments for second opinions—could enhance risk management by supporting more nuanced, personalized treatment plans that reconcile both aesthetic aspirations and functional goals, thereby reducing misunderstandings and medico-legal vulnerability.

### 4.3. Limitations

The relatively small number of studies included in the final analysis (*n* = 16) suggests that medico-legal aspects in orthodontics remain insufficiently explored in recent literature. Moreover, the studies originate from diverse regions, each with distinct healthcare and legal systems. These contextual differences can shape how medical harm is perceived and addressed, resulting in varying thresholds for conflict and litigation. It is also important to note that medico-legal data are often documented and monitored by professional organizations, regulatory authorities, and legal institutions, which serve as complementary sources of information beyond academic publications. This broader perspective provides valuable insights that are not always captured in scientific databases. While this heterogeneity may limit the generalizability of findings, it also highlights the need for continued research to better understand and address medico-legal risks in orthodontic practice.

## 5. Conclusions

According to the articles included in this review, orthodontics is often perceived as one of the dental specialties with the lowest medico-legal risk, as supported by the low incidence of malpractice complaints in most of the studies reviewed, even though we can see an increasing tendency in more recent studies. However, this apparent “occupational safety” should not lead to underestimating the inherent risks of orthodontic practice. The findings of this review emphasize that the most frequent malpractice allegations stem not from major clinical errors, but from *ethical–legislative*, *behavioral–relational*, and *perceptual–aesthetic vulnerabilities*—factors that foster conflict and increase reputational risks for practitioners.

A recurrent theme is the *information gap* between orthodontists and their patients, particularly in relation to the purpose and expectations of treatment. While the clinician prioritizes the functional correction of the dento-maxillary system, patients often focus on aesthetic outcomes. This misalignment highlights the importance of *recalibrating the doctor—patient relationship*, especially in the context of consent, communication, and expectation management.

Therefore, beyond clinical expertise, orthodontists must develop *transversal skills*—notably emotional intelligence and effective communication—to navigate the relational dimensions of care and prevent conflict. These abilities are crucial in a dynamic, increasingly complex healthcare environment where adaptability has become a key to both success and risk mitigation.

Finally, orthodontics is rapidly transitioning into a digitally assisted field, with technologies such as *AI*, *3D simulations*, *aligners*, and *teleorthodontics* shaping future practice. While these tools may offer enhanced precision and patient engagement, they also introduce new ethical and medico-legal considerations. This evolving digital landscape requires further research to ensure that innovation translates not only into clinical excellence, but also into greater litigation resilience and professional safety.

## Figures and Tables

**Table 1:** Information on the articles analyzed (author, year, country, subject).

Author	Year	Country	Subject	Ref.
Mizrahi E.	2010	United Kingdom	Risk Management	[6]
Umesan et al.	2012	Brunei	Risk Management	[7]
Franco et al.	2012	Brazil	Malpractice Claims	[8]
Kim YH, Hwang CJ.	2014	South Korea	Malpractice Claims	[9]
Ireland et al.	2015	United Kingdom	Risk Management	[10]
Abdelkarim A, Jerrold L	2015	USA	Risk Management	[4]
Abdelkarim A, Jerrold L	2015	USA	Risk Management	[11]
Tiro A.	2017	Bosnia and Herzegovina	Orthodontic’s Risks	[12]
Tiro A.	2018	Bosnia and Herzegovina	Orthodontic’s Risks	[13]
Makwakwa NL, Motloba PD	2019	South Africa	Orthodontics’ and other specialities’ malpractice claims	[14]
Laviv et al.	2020	Israel	Orthodontics’ Malpractice Claims	[15]
Picoli et al.	2020	Brazil	Orthodontics’ Malpractice Claims	[16]
Abomalik et al.	2022	Saudi Arabia	Orthodontics’ and other specialities’ malpractice claims	[17]
Pour et al.	2022	USA	Orthodontics’ Malpractice Claims	[18]
Zhao et al.	2024	China	Risk Management	[19]
Ramaswami et al.	2024	Brazil	Orthodontics’ and other specialities’ malpractice claims	[20]

**Table 2:** Frequencies of malpractice allegation types identified in the included studies.

Type of Allegation	Frequency (%)
Dissatisfaction with treatment outcomes	18.6–36.2
Periodontal issues	2.3–11.4
Root resorption	1.0–9.7
Prolonged treatment duration	12.9
Oral lesions and complications	20.3
Poor communication or insufficient explanations	32.0
Intervention by another orthodontist (second opinion)	25.0
Ethical and legislative issues (informed consent)	24.7–30.2

**Table 3:** The share of malpractice accusations in orthodontics, compared to other specialties.

Author	Country	Year	Subject	Ref.
Makwakwa et al.	South Africa	2019	1. Oro-maxillofacial surgery: 27.3% 2. Endodontics: 22.7% 3. Prosthetics: 22.7% 4. General dentistry: 9.1% 5. Periodontology: 6.8% ***6.*** ***Orthodontics: 4.5%*** 7. Unspecified: 6.8%	[14]
Ramaswami et al.	Brazil	2024	1. Oro-maxillofacial surgery: 27.5% 2. General dentistry: 20.9% 3. Endodontics: 15.4% 4. Prosthetics: 11.0% 5. Implantology: 11.0% ***6.*** ***Orthodontics: 8.8%*** 7. Pedodontics: 3.3% 8. Periodontology: 2.2%	[20]
Abomalik et al.	Saudi Arabia	2022	1. Prosthetics: 32.4% ***2.*** ***Orthodontics: 20.2%*** 3. Endodontics: 15.8% 4. Oral surgery: 13.2% 5. General dentistry: 8.8% 6. Implantology: 6.1% 7. Pedodontics: 2.6%	[17]

## Data Availability

No new data were created or analyzed in this study. Data sharing is not applicable to this article.

## Abbreviations

VUCA	Volatility, Uncertainty, Complexity, and Ambiguity
AI	Artificial Intelligence
AAO	American Association of Orthodontists
CAGR	Compound Annual Growth Rate
